# scDrug: From single-cell RNA-seq to drug response prediction

**DOI:** 10.1016/j.csbj.2022.11.055

**Published:** 2022-12-01

**Authors:** Chiao-Yu Hsieh, Jian-Hung Wen, Shih-Ming Lin, Tzu-Yang Tseng, Jia-Hsin Huang, Hsuan-Cheng Huang, Hsueh-Fen Juan

**Affiliations:** a Taiwan AI Labs, Taipei 10351, Taiwan; b Institute of Biomedical Informatics, National Yang Ming Chiao Tung University, Taipei 11221, Taiwan; c Department of Life Science, National Taiwan University, Taipei 10617, Taiwan; d Department of Computer Science and Information Engineering, National Taiwan University, Taipei 10617, Taiwan; e Graduate Institute of Biomedical Electronics and Bioinformatics, National Taiwan University, Taipei 10617, Taiwan; f Center for Computational and Systems Biology, National Taiwan University, Taipei 10617, Taiwan

**Keywords:** Single-cell RNA-seq, Drug repositioning, Bioinformatics, Tumor cell subpopulations

## Abstract

Single-cell RNA sequencing (scRNA-seq) technology allows massively parallel characterization of thousands of cells at the transcriptome level. scRNA-seq is emerging as an important tool to investigate the cellular components and their interactions in the tumor microenvironment. scRNA-seq is also used to reveal the association between tumor microenvironmental patterns and clinical outcomes and to dissect cell-specific effects of drug treatment in complex tissues. Recent advances in scRNA-seq have driven the discovery of biomarkers in diseases and therapeutic targets. Although methods for prediction of drug response using gene expression of scRNA-seq data have been proposed, an integrated tool from scRNA-seq analysis to drug discovery is required. We present scDrug as a bioinformatics workflow that includes a one-step pipeline to generate cell clustering for scRNA-seq data and two methods to predict drug treatments. The scDrug pipeline consists of three main modules: scRNA-seq analysis for identification of tumor cell subpopulations, functional annotation of cellular subclusters, and prediction of drug responses. scDrug enables the exploration of scRNA-seq data readily and facilitates the drug repurposing process. scDrug is freely available on GitHub at https://github.com/ailabstw/scDrug.

## Graphical abstract

**Figure:**
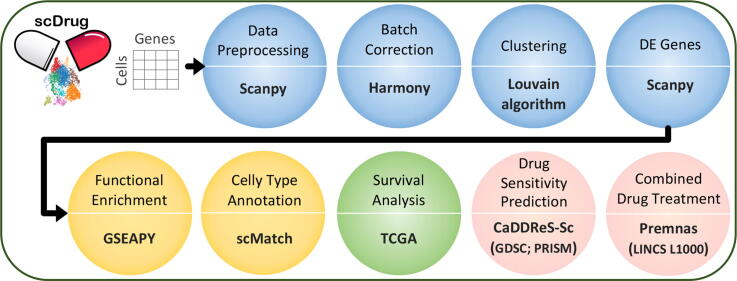

## 1 Introduction

Analyses of single-cell RNA sequencing (scRNA-seq) datasets have become more commonly used to profile high-resolution cellular composition, leading to the discovery of tumor heterogeneity [1] and offering an unprecedented opportunity to study specific biological questions [2], [3]. Recently, several scRNA-seq studies have identified individual cell types, even in tumor tissues containing complex cell populations [4]. Thus, the details of the expression signature of the malignant tumor cells provide excellent targets for identifying suitable drug treatments. Additionally, targeting malignant tumor sub-cell types may prolong patients’ survival.

Recent years have seen the explosion of research into scRNA-seq and its applications in clinical practice. For example, cancer patients can benefit from the most effective medical treatment by analyzing the scRNA-seq data from the tumors [5]. For example, the integration of scRNA-seq data and the drug response profiles of the cancer cell lines from the Library of Integrated Network-based Cellular Signatures (LINCS) [6] can successfully select effective drugs to target specific cell subpopulations [7]. In addition, large-scale projects such as the Cancer Genome Atlas Program (TCGA) [8], Cancer Cell Line Encyclopedia (CCLE) [9], the Genomics of Drugs Sensitivity in Cancer (GDSC) [10], and the LINCS [6], have provided valuable datasets to link the gene expression and drug treatments.

Drug repositioning is an effective strategy for approved or investigational drugs to develop new treatments for a different disease [11]. Several studies have developed different prediction models based on multi-omic data for the selection of potential drugs in cancer [12], [13], [14]. Further, recent studies have leveraged scRNA-seq and bulk transcriptomic profiles to predict drug response for heterogeneous tumor cells, such as Beyondcell and CaDRReS-Sc [15], [16]. However, incredible numbers of scRNA-seq analysis tools make a difficult learning curve for any biomedical researcher or clinician to readily explore scRNA-seq datasets for translation.

With the maturation of scRNA-seq and bioinformatics analysis, vast numbers of analytical tools have been developed in the past years. Therefore, we develop a new tool, scDrug, from scRNA-seq analysis to drug response prediction. In scDrug, we first constructed the workflow, the scRNA-seq analysis pipeline, for a comprehensive analysis of the scRNA-seq data. scDrug provides an easy-to-use pipeline with scRNA-seq data analysis to sub-clustering tumor cells under a Python environment. Next, we integrated two different approaches to predict drug treatments to target cancer cell subpopulations using public datasets for comprehensive molecular and pharmacological characterization of cancer cell lines, including LINCS [17], GDSC [10] and PRISM [18]. Specifically, one approach predicts drug sensitivity to a specific tumor cluster, and the other predicts the combined effect of drugs on tumor clusters. Herein, scDrug faithfully provides the prediction results for the domain experts to evaluate the selected drugs. Unlike BeyondCell [15] that assesses cell clusters based on drug sensitivity scores, scDrug applies the conventional method using gene expression profiles to annotate tumor clusters. Our validation results demonstrated that scDrug could successfully capture cell responses to drug treatments. scDrug allows researchers to explore the heterogeneity of tumor cells and suggests candidate drugs for effective treatments.

## 2 Materials and methods

### 2.1 scDrug scRNA-seq data preprocessing

The first step in the pipeline is scRNA-seq data analysis, including data preprocessing by Scanpy [19], imputation by MAGIC [37], batch correction by Harmony [20], clustering by Louvain algorithm [21], identification of differentially expressed genes (DEGs) by Scanpy [19], functional enrichment by GSEAPY [22], [23] and cell-type annotation by scMatch [24].

In data preprocessing, we filtered out cells with less than 200 genes expressed and genes expressed in less than three cells and kept the cells with a proportion of mitochondrial genes below 30 %. The remaining data undertook normalization to 10,000 total counts per cell, natural logarithmic transformation, highly variable gene annotation, and scaling to unit variance and zero means. Once the data imputation is needed, scDrug also integrates MAGIC [37] to impute missing values. Next, we applied principal component analysis (PCA) and adjusted the principal components (PCs) with the Harmony algorithm to eliminate batch effects if needed. We then computed a neighborhood graph on the top 20 PCs and used the Louvain algorithm to cluster cells into groups.

### 2.2 scDrug auto-resolution for clustering

To determine the resolution for clustering, users can choose from manual or automatic assignment. In the automatic mode, we calculated the subsampling-based robustness score described in chooseR [25] for resolution values in the interval [0.4, 1.4] with 0.2 spacing. For a given resolution, the average silhouette scores [26] were calculated using the distance matrix defined as 1.0 subtracting the co-clustering frequency of 5 repetitions of the clustering, each performed on a random 80 % subset of the dataset, drawn without replacement. We regarded the resolution with the highest score as the optimal clustering resolution.

### 2.3 Differential gene expression analysis, cell annotation, and functional enrichment

After clustering, scDrug ranked the genes for each cluster to identify differentially expressed genes (DEGs) by using scanpy function *rank_genes_groups* with default parameters. Then, scDrug performed functional enrichment with GSEAPY [22], [23]. In addition, we used the human GO_Biological_Process_2021 library to execute Enrichr [22] on DEGs with log2 fold change above 2 and *p*-value and adjusted *p*-value both below 0.01. For cell-type annotation, we used the expression of all genes and calculated the mean expression of its cells as its gene expression profile (GEP) for each cluster. Next, we applied scMatch [24] to annotate cluster-wise cell types based on their GEPs from the truncated FANTOM5 reference dataset [27].

Based on the output of scRNA-seq data analysis, including a scanpy AnnData object [19], a gene expression profile (GEP), UMAPs of the results of batch correction, clustering, and cell-type annotation, and files of DEGs and GSEA [23], users can apply sub-clustering for further inspection by repeating this single-cell data analysis procedure on specified clusters of previously produced AnnData.

### 2.4 Survival analysis

To predict how each cluster will affect patients’ survival, we applied the method proposed by Lin et al. [28]. First, we selected each cluster’s top 20 differentially expressed genes as a set of cluster-specific gene signatures. Then, the bulk RNA profiles and the corresponding clinical information for patients with different cancer were downloaded from the TCGA database [29]. To evaluate the tumor cluster activity in each patient, we constructed an expression table for each patient, with each column representing the gene signature of a cluster. For each cluster and each of its 20 selected genes, the value is assigned to 1 if the gene expression in the patient is higher than its median expression in all the patients; otherwise, the value is set to 0. The column-wise sum (hereafter referred to as the “activity score”) indicates the activation level of each cluster in the patient. For each cluster, patients were divided into “high-expressing” and “low-expressing” groups if their activity score for that cluster was in the top or bottom quartile. Finally, we compared the survival of these two groups with the Kaplan–Meier curves and *p*-value of the log-rank analysis (Supplementary Fig. S1).

### 2.5 Drug response prediction

In the scDrug pipeline, we used the AnnData object generated in the first step (scRNA-seq data analysis) and applied CaDRReS-Sc [16] for drug response prediction. CaDRReS-Sc is a machine-learning framework for robust cancer drug response prediction based on scRNA-seq data, which estimated cell clusters' half-maximal inhibitory concentration (IC_50_). Based on the CaDRReS-Sc framework, we provide two pre-trained prediction models, GDSC and PRISM, for the drug response of cell clusters.

The two models were trained using the gene expression and drug response data from the GDSC and PRISM datasets via the objective function without sample bias. We evaluated the prediction performance by calculating drug-wise Spearman correlation coefficients with actual and predicted drug response values. In ascending order, we dropped the drugs whose drug-wise coefficient was lower than the first quartile coefficient.

#### 2.5.1 Drug-response training data

For the GDSC model, we used the response data (measured IC_50_) of 226 drugs in 1074 cancer cell lines, provided by CaDRReS-Sc from the GDSC database, as our training data. For the PRISM model, we used the PRISM Repurposing dataset (version 19Q4) as the training data, which contained the responses of 1448 drugs against 480 cell lines. Instead of IC_50_, the PRISM dataset provides the drug response in terms of the area under the dose–response curve (AUC). The drug response values were on different scales in two datasets showing an overall Pearson correlation coefficient of 0.615 between the PRISM values (1 − AUC) and the GDSC values (−log(IC_50_)) (Supplementary Fig. S2). To accommodate the CaDRReS-Sc model framework and increase its prediction power, we adopted scaled 1 - AUC as drug response values. The scaling formula was defined as

$$D^{\prime }=240D-120,$$

where D′ and D represent scaled and unscaled 1 - AUC, respectively. Evaluation of different scaling coefficients is shown in Supplementary Fig. S3.

#### 2.5.2 Gene-expression profiles as features

For the GDSC model, we used the gene expression data of 1,018 cancer cell lines provided by CaDRReS-Sc from the GDSC database, and selected the 17,419 common genes among all cell lines as feature genes for model training. For the PRISM model, the CCLE (Cancer Cell Line Encyclopedia) expression data (version 21Q3) was downloaded from DepMap Portal (https://depmap.org/portal/) and contained 1,379 cell lines and 19,177 genes. We used 8,087 genes whose expressions were correlated with PRISM AUC with at least 0.2 absolute Pearson correlation coefficient as features genes. We calculated log_2_ expression fold-change for average expression across cell lines for each feature gene. The kernel feature is cell–cell similarity using the Pearson correlation coefficient based on this fold-change profile.

#### 2.5.3 Prediction model framework

To predict the IC_50_ of cell clusters, we computed the log_2_ fold change with respect to the average gene expression values of AnnData and predicted the IC_50_ value for each cell. The mean IC_50_ prediction then determines the IC_50_ of cell clusters among the cells within each cluster. Alternatively, we utilized the log2 fold change between a cluster and others and predicted the cluster-wise IC_50_ directly. We modified the CaDRReS-Sc method for the PRISM model by replacing the learning variable IC_50_ with scaled 1 - AUC. The model learned latent pharmacogenomic relations from the transcriptomic and drug response profiles. The cancer cluster-specific model proposed in CaDRReS-Sc [16] was defined as

$${\hat{s}}_{\mathrm{iu}}=\mu +b_{i}^{Q}+b_{u}^{P}+q_{i}∙p_{u}=\mu +b_{i}^{Q}+b_{u}^{P}+q_{i}{\left(x_{u}W_{P}\right)}^{T}$$

where $s_{\mathrm{iu}}$ is the observed drug response (IC_50_ or scaled 1 - AUC) of cell line u to drug i, ${\hat{s}}_{\mathrm{iu}}$ is the predicted drug response, μ is the overall mean drug response, $b_{i}^{Q}$ and $b_{u}^{P}$ are the bias terms for drug i and cell line u, respectively, $q_{i},p_{u}\in R^{f}$ represent drug i and cell line u in the f-dimensional latent space, $W_{P}\in R^{d\times f}$is a transformation matrix that projects cell line features (gene expression levels) $x_{u}\in R^{d}$ onto the latent space, and d is the number of genes.

The bias terms, $b_{u}^{P}$ and μ, don’t capture the true bias from unseen cluster, so they were removed, and the objective function was defined as

$$L\left(\theta \right)=\frac{1}{2K}\left[\sum_{i}\sum_{u}{\left(s_{\mathrm{iu}}-{\hat{s}}_{\mathrm{iu}}\right)}^{2}+\lambda \sum_{d}{\|w_{d}\|}^{2}+\lambda \sum_{i}{\|q_{i}\|}^{2}\right]$$

$${\hat{s}}_{\mathrm{ui}}=b_{i}^{Q}+q_{i}∙p_{u}$$

where K is the total number of drug-cell pairs, λ is the L2-regularization parameter, and $w_{d}$ is a vector in $W_{P}$. A diagram of the detailed prediction procedure is shown in Supplementary Fig. S4.

#### 2.5.4 Model training and evaluation

The PRISM and GDSC models were trained with 140-dimensional and 10-dimensional latent space, respectively, with a learning rate of 0.01 and maximum epochs set to 100,000. To evaluate the performance of unseen cell lines, we split 24 cell lines as the validation set and calculated their median absolute error and Pearson correlation coefficient between actual and predicted drug response.

### 2.6 Combined treatment prediction

We adopted a recently published computational framework, Premnas [30], to predict potential combined treatment strategies using the LINCS L1000 data [17]. Firstly, we generated bulk GEPs from the LINCS L1000 database of a user-specified or automatically determined reference cell line. Eight cell lines with a substantial number of drug perturbation experiments, including A375 (malignant melanoma), A549 (non-small cell lung carcinoma), HCC515 (non-small cell lung adenocarcinoma), HEPG2 (hepatocellular carcinoma), HT29 (colorectal adenocarcinoma), MCF7 (breast adenocarcinoma), PC3 (prostate adenocarcinoma), and YAPC (Pancreatic carcinoma), were used in the scDrug. As for the cell type assignment, we calculated the Pearson correlation coefficient between the single-cell GEP and the bulk GEP of each reference cell type and selected the one with the highest correlation. The anti-logarithmic gene expression profiles of all experiments tested on this cell line were the bulk GEPs used for cell distribution inference afterward.

After extracting the corresponding GEPs from the LINCS L1000 database, we estimated the proportion of each cell subset in bulk samples by CIBERSORTx [31]. Subsequently, we assessed the differences in the cell-subset distribution of treated and control samples for each perturbation. The cell subsets whose percentage is reduced more than 90 % after perturbation were considered “killed” by that perturbation. Also, we set a consistency threshold to strengthen the authenticity of the drug efficacy; that is, the reduction (default: 75 %) of the “killed” subset should also be observed in the perturbations using the same compounds at higher concentrations. Finally, we employed a greedy search strategy to provide a combined treatment that inhibits the growth of the greatest number of tumor subpopulations. Briefly, we selected the perturbations that can kill the highest number of subpopulations and have the greatest efficacy by the sum of the reductions across all subpopulations. Of note, the perturbations which result in an identical number of killed subpopulations and the effects were used separately to suggest more combinations. For each iteration, the killed subpopulations were removed, and we recursively added perturbations one at a time until no more subpopulations could be killed.

## 3 Results

### 3.1 Workflow of scDrug pipeline

**Fig. 1:**
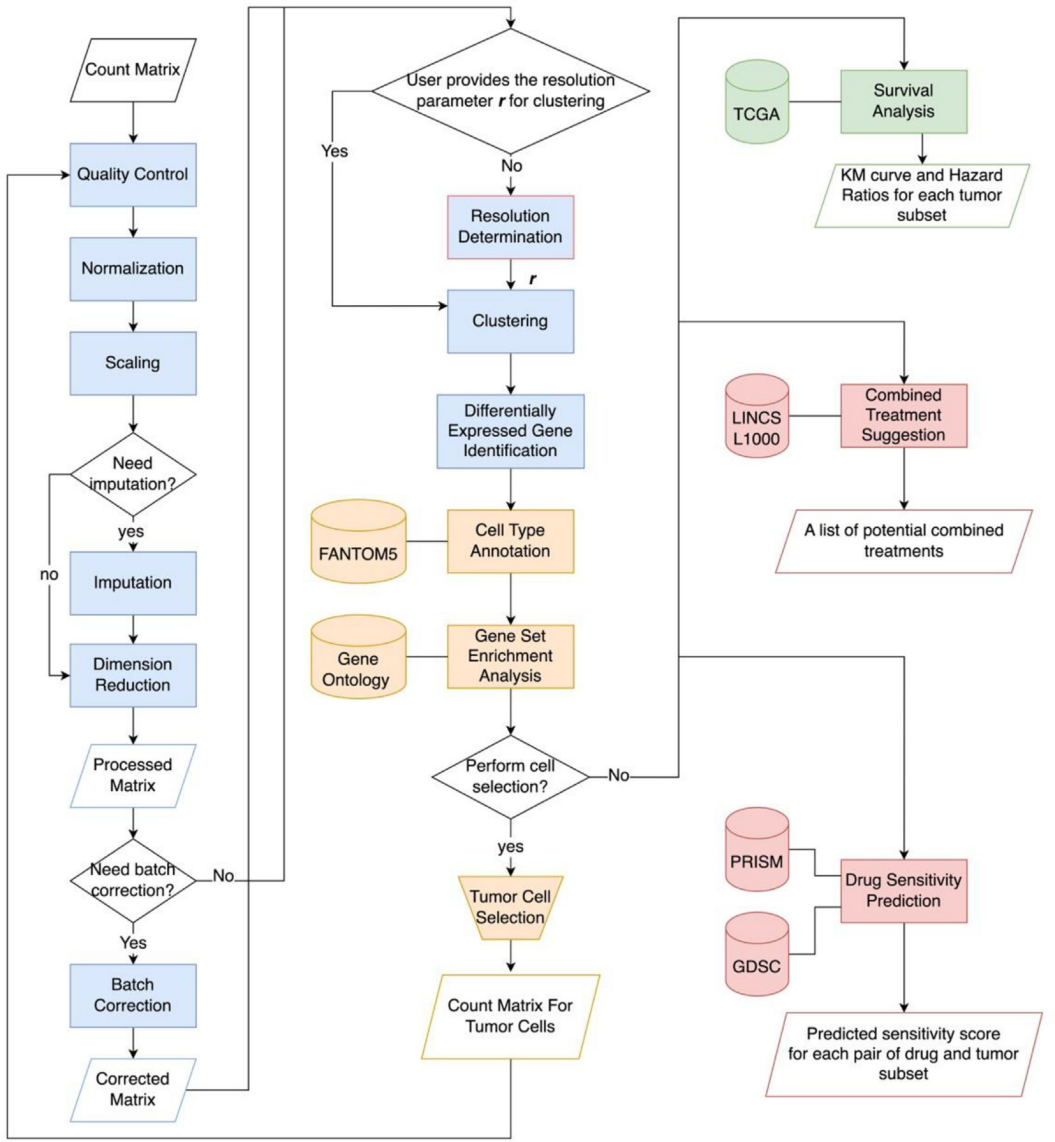
Workflow of scDrug. The first part of scDrug analyzes the scRNA-seq to generate the cell clusters (blue). The second part of scDrug performs the cell type and functional annotations (yellow). The third part of scDrug runs the survival analysis to help identify malignant tumor cell clusters (green) and finally predict candidate drugs with two different methods (red). (For interpretation of the references to colour in this figure legend, the reader is referred to the web version of this article.)

The workflow of the scDrug pipeline consists of three main modules: 1) scRNA-seq analysis for identification of tumor cell subpopulations, 2) functional annotation of cellular subclusters, and 3) drug repositioning prediction (Fig. 1).

### 3.2 Validation of scDrug pipeline

**Fig. 2:**
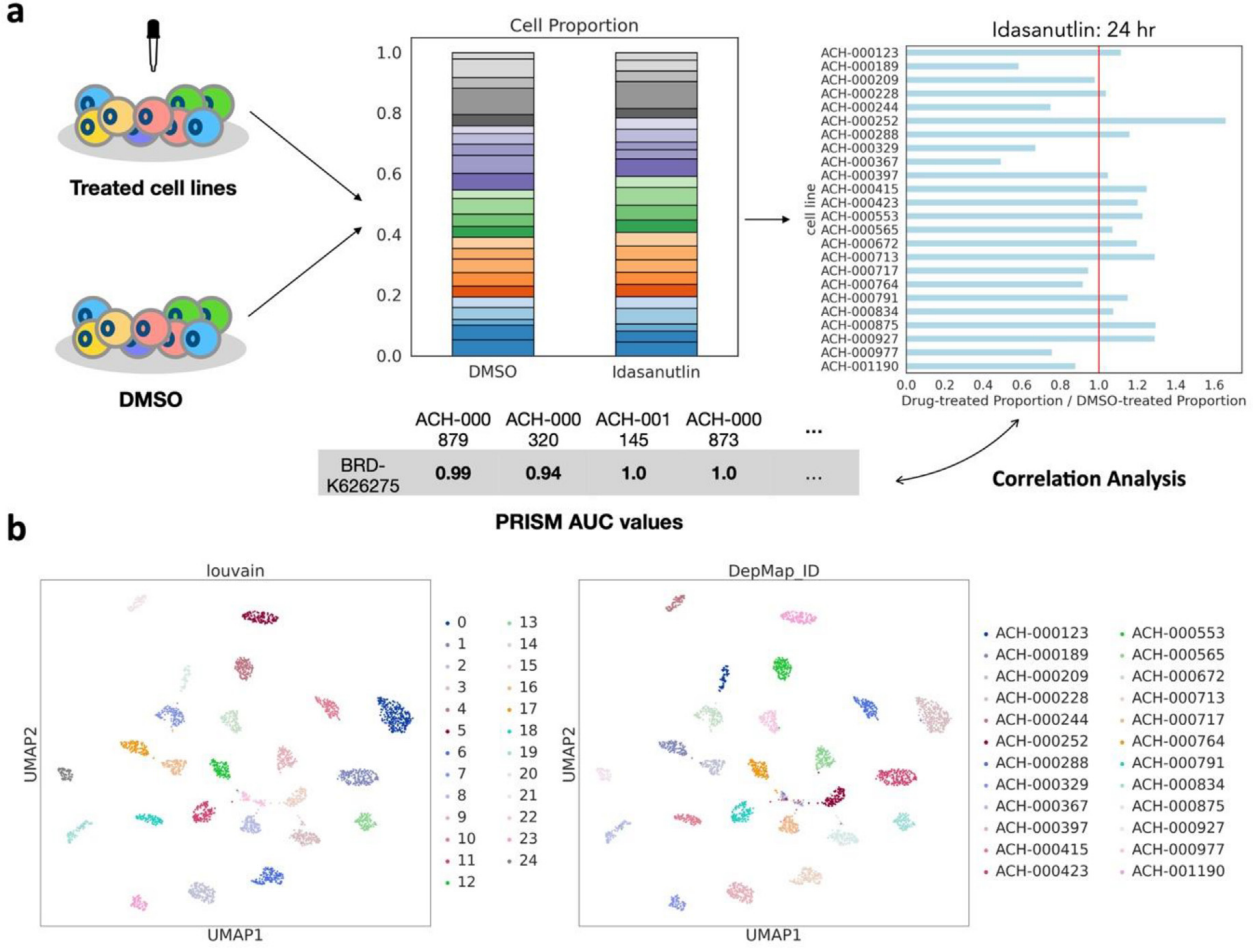
Validation of scDrug pipeline. (a) Schema of validating drug-sensitivity predictions. First, scDrug successfully identified all 24 cell lines in both treated and untreated datasets after preprocessing and clustering. Next, the cell proportion change of a cell line was calculated as the ratio of its percentage in the treated data to its ratio in the untreated data. The proportion changes for all cell lines were then compared to the corresponding predicted sensitivity scores using Spearman's correlation coefficient analysis. (b) UMAP visualization for Idasanutlin-treated cells. The clustering result colored the left, and the right was colored by the DepMap IDs provided by McFarland et al. [32].

To validate our scDrug pipeline for predicting cell sensitivity, we used the scRNA-seq data for cells originating from 24 distinct cell lines treated with idasanutlin for 24 h [32]. The results showed that scDrug could (1) distinguish all 24 cell lines and (2) accurately predict their corresponding sensitivity to idasanutlin (Fig. 2).

For both the treated and the control (the one treated with DMSO) datasets, scDrug processed the raw 10X data as described before, and it discovered all the 24 cell lines that each were identified as a single cluster and one extra cluster mixed with a variety of cells in both datasets. We removed the mixed cluster for the downstream analysis. Next, we calculated the fold change in the number of cells per cell line after treatment. Afterward, scDrug estimated each cell line's sensitivity to idasanutlin. The model pre-trained by the control and PRISM data was used to predict the sensitivity score for each cluster (cell line). As expected, the proportion changes were negatively correlated with the predicted sensitivity scores significantly (Spearman's correlation coefficient = −0.42 with *p*-value = 0.04), indicating that scDrug can reflect the drug sensitivity for different cell types.

## 4 Case studies

### 4.1 Application example I. Hepatocellular carcinoma

**Fig. 3:**
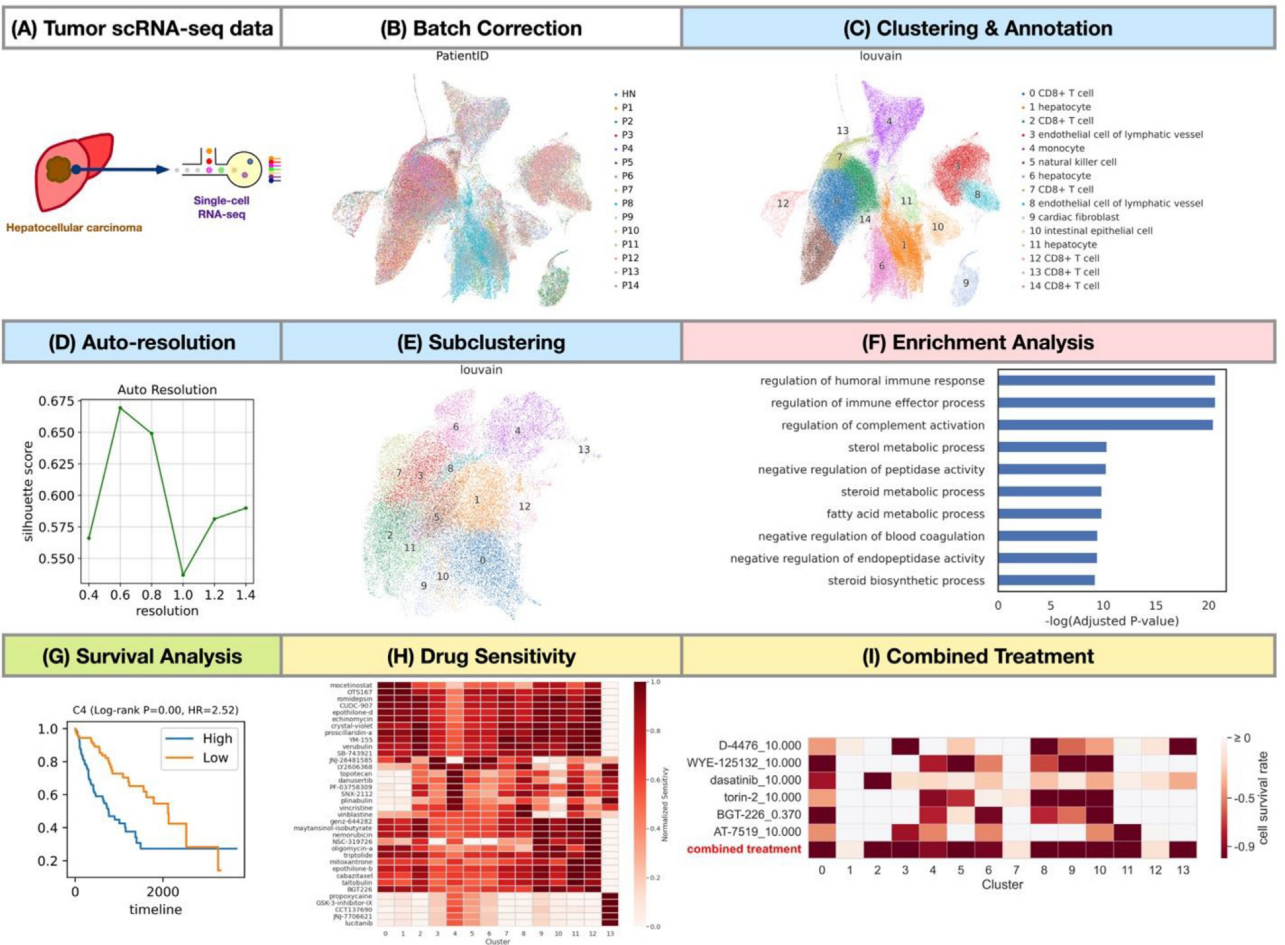
Case study on hepatocellular carcinoma. (A) The scRNA-seq of Hepatocellular carcinoma was obtained from Sharma et al. (2020); (B) UMAP for patientID distribution after Harmony batch correction; (C) UMAP for the cellular clustering; (D) Auto-resolution is performed by scDrug; (E) Sub-clustering for potential tumor cells from (C); (F) Gene set enrichment analysis for gene ontology annotation of a cell cluster; (G) An example of KM curve for survival analysis; (H) Heatmaps showing the potential drugs in the PRISM database predicted to inhibit cell growth by CaDRReS-Sc. Each cell represents values of predicted sensitivity score of the tumor cell clusters to the drugs; (I) Heatmap showing the optimal combined treatment of six drugs in the LINCS L1000 database to kill the greatest number of cell clusters according to the Premnas prediction.

We demonstrate the application of the scDrug pipeline on the hepatocellular carcinoma scRNA-seq data (GSE156625), which comprises tens of thousands of cells from patient tissues [33]. We performed the scDrug pipeline to recognize the tumor sub-clusters and then predict the candidate drugs for targeting tumor cells (Fig. 3).

From the hepatocellular carcinoma scRNA-seq raw data (Fig. 3A), we first conducted data analysis and biological annotation to identify the tumor sub-population. First, we filtered out the genes and cells with low expression and poor-quality cells showing a high percentage of mitochondrial genes. On the remaining 106 thousand cells, we selected 2307 out of 25,314 highly variable genes for further analysis. Then, we applied PCA to reduce the high dimensionality of single-cell data. Since the cells were collected from several patients, we eliminated the batch effects caused by different patients using the Harmony algorithm (Fig. 3B). Next, Louvain clustering analysis resulted in 15 cell groups and we annotated each cluster a cell type by using the FANTOM5 database (Fig. 3C). Among the 15 cell clusters, we considered the 3 clusters with tumor cell percentages over twice the normal cell percentages as the tumor clusters. Then, we performed sub-clustering on the tumor subpopulation at an automatically determined resolution of 0.6 (Fig. 3D), generating 14 sub-clusters (Fig. 3E). After clustering, we identified the cluster DEGs and performed the GSEA (Fig. 3F).

Survival analysis was then applied to identify the most malicious cells among the 14 potential tumor subpopulations. Bulk RNA-seq data samples with hepatocellular liver carcinoma (LIHC) were downloaded from the TCGA. For each tumor subpopulation identified by the single-cell RNA-seq data, we divided the TCGA-LIHC samples into two groups by assessing the expression of its cell signature genes to perform the Kaplan–Meier curves and the log-rank test analysis. We found that the Cluster 4-specific gene signature was significantly associated with shorter survival (hazard ratio = 2.52; −log_2_(*p*-value) = 14) (Fig. 3G, see the supplementary Table S1 for the top 20 genes in 14 clusters). Scientists have reported that PTMA and STMN1, the top 2 DEGs of Cluster 4, are related to poor prognosis in hepatocellular carcinoma [34], [35].

Moreover, based on the sub-clustering outcome, we predicted the drug response and the combined treatments for the tumor subpopulation using the LINCS and PRISM databases. The top-ranked candidate drugs from the PRISM database are shown in Fig. 3H and the sensitivity values of all drugs for different sub-clusters are listed in Supplementary Table S2. For the treatment suggestions from the LINCS L1000 database, scDrug suggests a combined treatment with a minimal therapeutics combination capable of killing 11 out of 14 sub-clusters effectively (Fig. 3I, Supplementary Table S3).

### 4.2 Application example II. Prostate cancer

We also analyzed the scRNA-seq data for prostate cancer [36] with scDrug. Same as in the processing steps described in application example I, scDrug has removed low-quality cells, reduced the batch effects, and performed clustering that the number of clusters was determined by the highest silhouette score instead of a predefined number (the best number of clusters is 15), and annotated each cluster a known cell type by comparing the average gene expression between that cluster and each of 916 cell types in the FANTOM5 database. Finally, among 17,092 cells in the prostate cancer dataset, we identified 15 cell types, including normal immune cells and potential tumor cells.

**Fig. 4:**
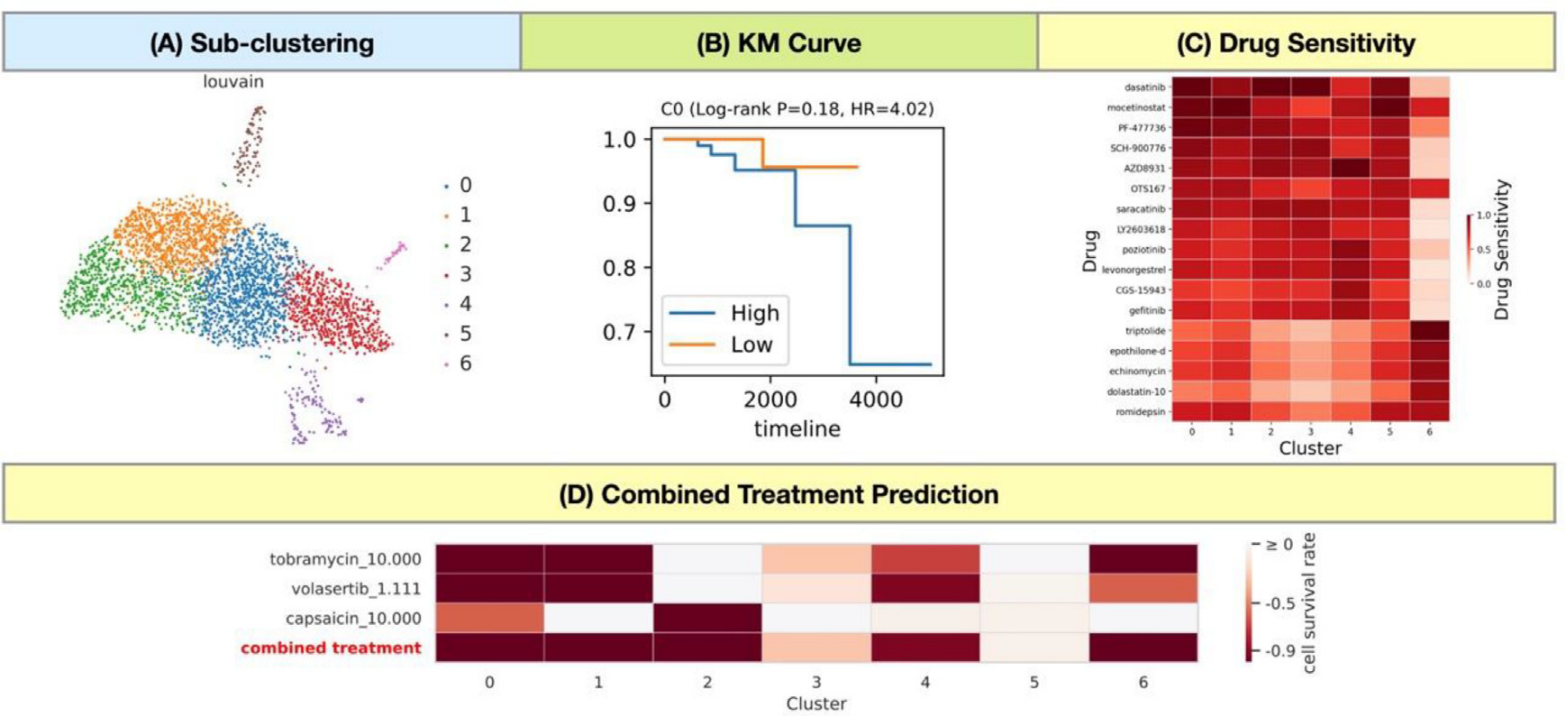
Case study on prostate cancer. (A) The scRNA-seq of prostate cancer was obtained from Tuong et al. (2021). Sub-clustering for potential tumor cells according to the auto-resolution at 0.6; (B) An example of KM curve for survival analysis; (C) Heatmap showing the optimal combined treatment of six drugs in the LINCS L1000 database to kill the greatest number of cell clusters according to the Premnas prediction; (D) Heatmaps showing the potential drugs in the PRISM database predicted to inhibit cell growth by CaDRReS-Sc. Each cell represents values of the predicted sensitivity score.

Six clusters (3458 cells in total) with twice as many cells from tumor tissue as from normal tissue were defined as tumor cells and were pooled together to re-analyze. The scDrug classified these tumor cells into seven clusters with the auto-resolution function (Fig. 4A). In the survival analysis, cluster 0 was identified as one of the most harmful clusters that could lead to an unfavorable prognosis by the survival analysis (Hazard Ratio = 4.02; log-rank *p*-value = 0.18) (Fig. 4B). Notably, none of tumor clusters in this dataset yielded a statistical significance at *p*-value <0.05 for the survival analysis. Some possible reasons of this result were due to poor alignment of expression profiles between the heterogenous scRNA-seq dataset and the TCGA data, which has limited sample numbers and cancer types. For the drug repurposing prediction, most of the tumor clusters are sensitive to several drugs, such as dasatinib and mocetinostat (Fig. 4C, full list is shown in Supplementary Table S4) Moreover, the combinations of tobramycin, volasertib, and capsaicin enable to a reduction of more than 90 % of cells in cluster 0 and the combination of three drugs are effective to kill 5 out of 7 tumor clusters (Fig. 4D, detail information in Supplementary Table S5).

## 5 Conclusion

scDrug is a user-friendly pipeline capable of analyzing scRNA-seq data for drug discovery by integrating various bioinformatics tools. We demonstrated that our package could be used to study tumor scRNA-seq data and aid researchers in combating cancers with the sophisticated suggestion in drug selection. The prediction of drug response in scDrug is limited by experimentally screened compounds, and the available scRNA-seq data with drug treatments are short for evaluation. Future studies on *de novo* prediction of drug sensitivity and extensive experimental validations of the pipeline are necessary. scDrug is in continuous development and open to community contributions.

## Funding

This work was supported by the 10.13039/100007225Ministry of Science and Technology (MOST 109-2221-E-002-161-MY3, MOST 109-2221-E-010-012-MY3, MOST 109-2327-B-002-009, and MOST 111-2321-B-002-017), the Higher Education Sprout Project of Ministry of Education (NTU-111L8808, NTU-CC-109L104702-2, NTU-CC-111L893302) in Taiwan.

## CRediT authorship contribution statement

**Chiao-Yu Hsieh:** Methodology, Software, Data curation, Investigation, Visualization, Writing – original draft. **Jian-Hung Wen:** Methodology, Software, Data curation, Investigation, Visualization, Writing – original draft. **Shih-Ming Lin:** Data curation, Software. **Tzu-Yang Tseng:** Investigation, Data curation. **Jia-Hsin Huang:** Visualization, Funding acquisition, Investigation, Project administration, Writing – review & editing. **Hsuan-Cheng Huang:** Methodology, Visualization, Conceptualization, Supervision, Writing – review & editing, Funding acquisition. **Hsueh-Fen Juan:** Conceptualization, Supervision, Writing – review & editing, Funding acquisition.

## Declaration of Competing Interest

The authors declare that they have no known competing financial interests or personal relationships that could have appeared to influence the work reported in this paper.
