# Supplementary material for: scDrug: From single-cell RNA-seq to drug response prediction
Source: Comput Struct Biotechnol J. 2022 Dec 1;21:150–7. doi: 10.1016/j.csbj.2022.11.055 (PMC9747355; doi:10.1016/j.csbj.2022.11.055)
Supplement: Supplementary data 1 [file mmc1.docx]

scDrug: From single-cell RNA-seq to drug response prediction

Chiao-Yu Hsieh^1^, Jian-Hung Wen^1,2^, Shih-Ming Lin^1,3,4^, Tzu-Yang Tseng^3,5^, Jia-Hsin Huang^1,*^, Hsuan-Cheng Huang^2,*^ and Hsueh-Fen Juan^1,3,5,6,*^

^1^Taiwan AI Labs, Taipei 10351, Taiwan, ^2^Institute of Biomedical Informatics, National Yang Ming Chiao Tung University, Taipei 11221, Taiwan, ^3^Department of Life Science, ^4^Department of Computer Science and Information Engineering, ^5^Graduate Institute of Biomedical Electronics and Bioinformatics, ^6^Center for Computational and Systems Biology, National Taiwan University, Taipei 10617, Taiwan.

*To whom correspondence should be addressed. [jiahsin.huang@ailabs.tw](mailto:jiahsin.huang@ailabs.tw) (JHH); [hsuancheng@nycu.edu.tw](mailto:hsuancheng@nycu.edu.tw) (HCH); [yukijuan@ntu.edu.tw](mailto:yukijuan@ntu.edu.tw) (HFJ)

Supplementary Figures


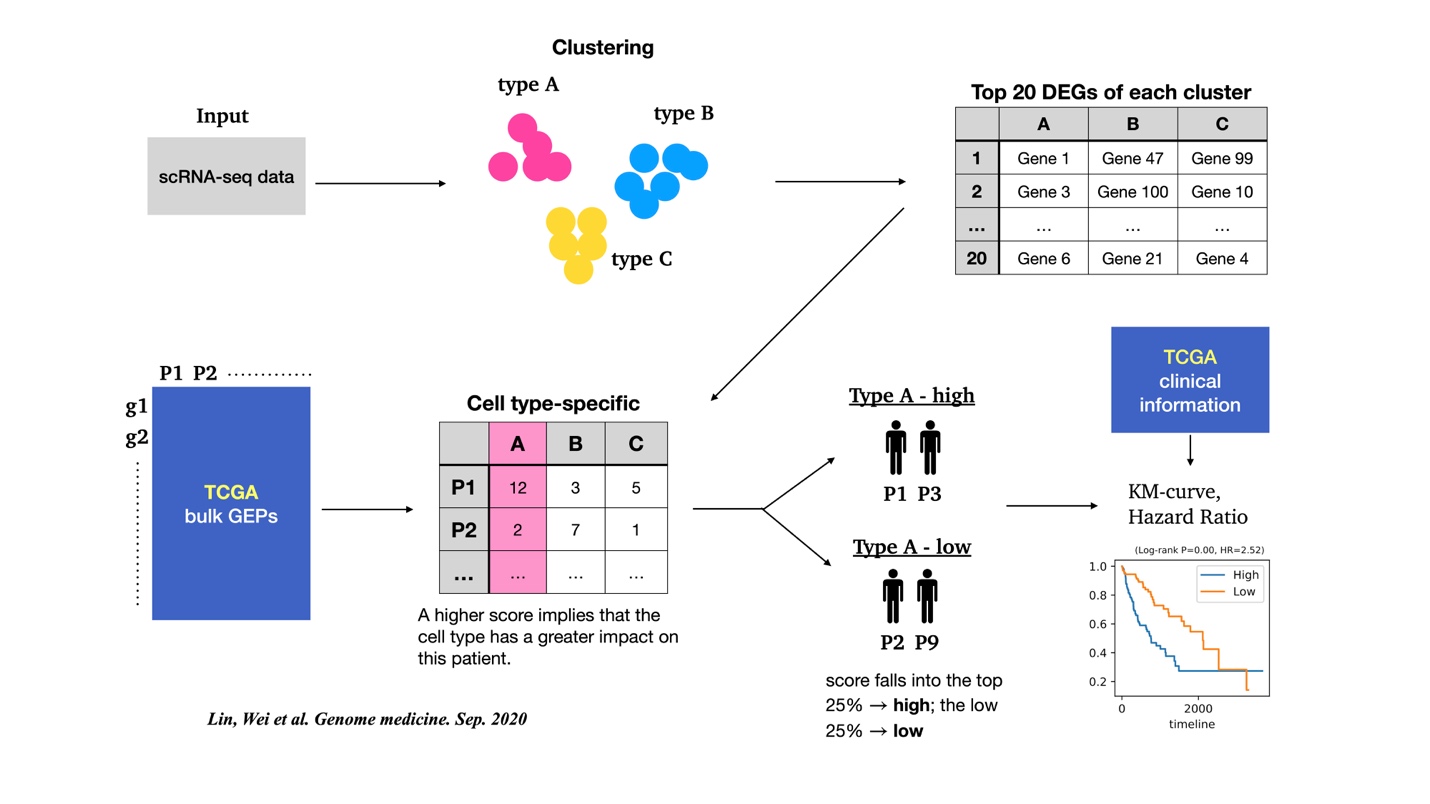


**Supplementary Figure S1. Illustration for survival analysis**. From the TCGA samples, we assessed the expression levels of each cell-type-specific signature identified by the scRNA-seq data. For each cluster (cell type), we divided patients into two groups, one overexpressing its signature and the other underexpressing the same signature. Finally, we compared the survival of these two groups with the Kaplan–Meier curves and log-rank p-value analysis.


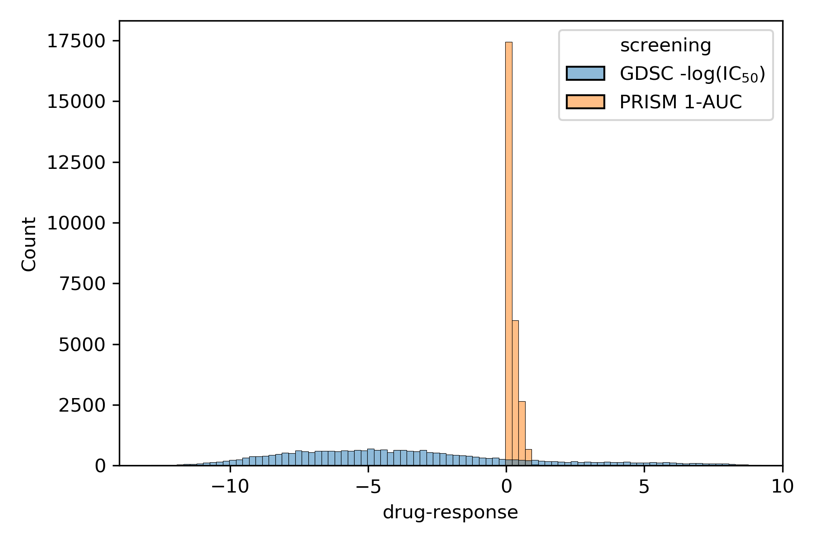


**b**

**a**


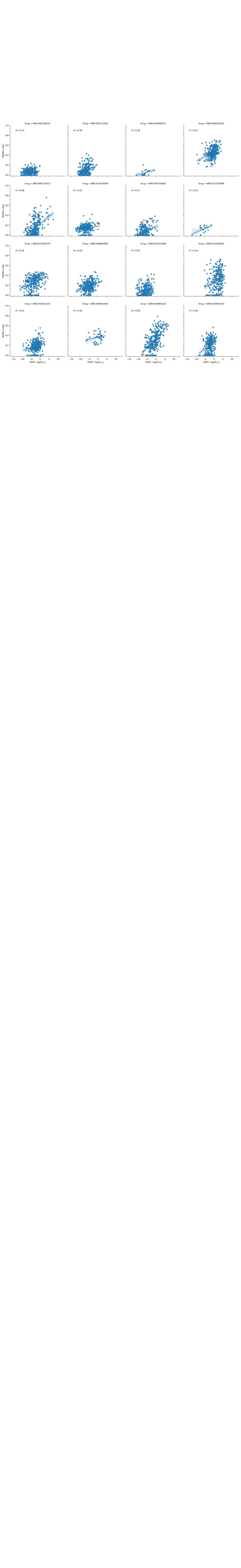


**Supplementary Figure S2. The differences between PRISM and GDSC.** (**a**) The histogram shows the scales of overall drug response values in two datasets are different. Each count represents a drug-cell-line pair. (**b**) Examples for demonstrating the screening results in PRISM and GDSC are different. Each dot in the scatter plots represents a cell line screened in both datasets for one drug. R is the Pearson correlation coefficient.


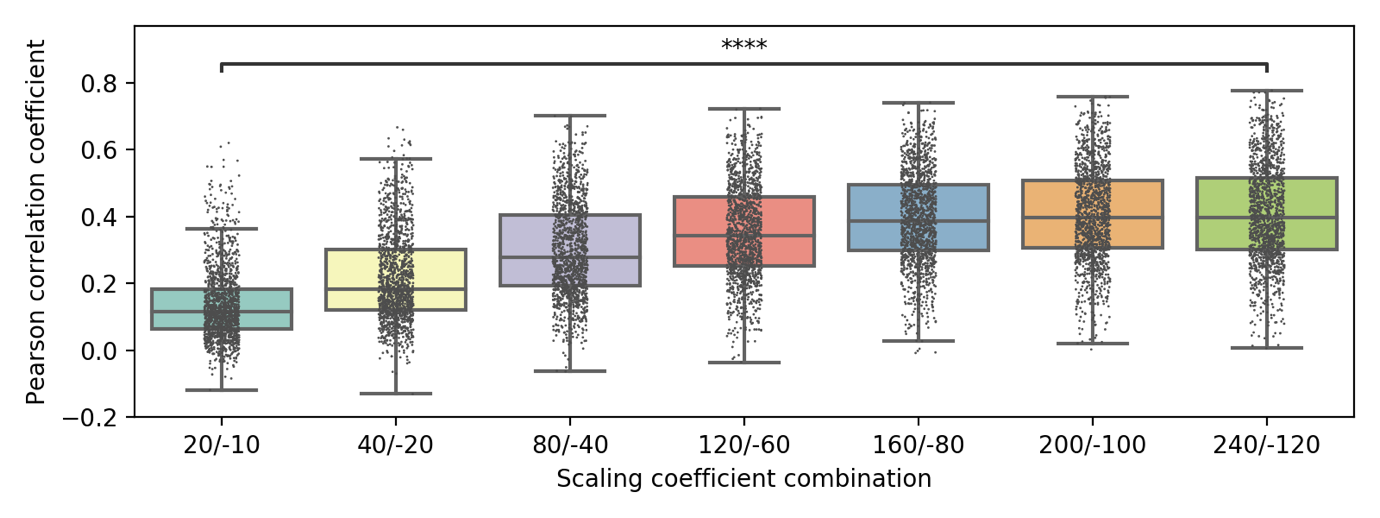


**Supplementary Figure S3. Drug-wise Pearson correlation coefficients between the predicted and the ground truth 1**$-$**AUC with different scaling coefficient settings.** The x-axis labels represent the scaling coefficients, $a$, $b$ in equation $D^{'}=aD+b$, in the format of $a/b$. The scaling coefficients were initially set as $a=20, b= -10$ since −log(IC50) values are mostly between $10$ and $-10$ and $1-$ AUC ranges from 0 to 1 (Supplementary Figure S2). Each dot represents the Pearson correlation coefficient of a drug. Statistical significance was performed by Wilcoxon rank-sum test with FDR correction. (ns: non-significant, *: $0.01<p\leq0.05$, **: $0.001<p\leq0.01$, ***: $0.0001<p\leq0.001$, ****: $p\leq0.0001$)


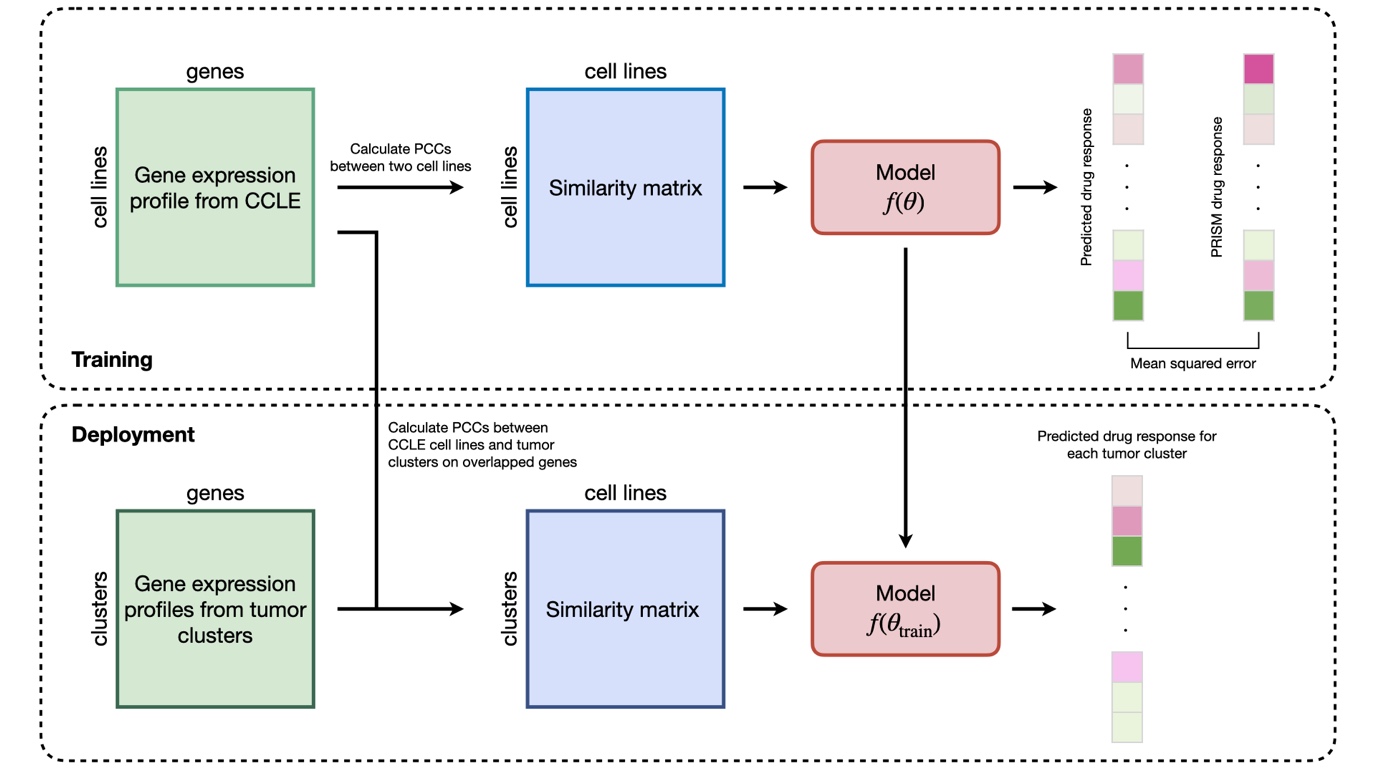


**Supplementary Figure S4. A diagram shows training a model for PRISM drug response and using it for downstream application.** For the training task, the objective is to minimize the loss between predicted and ground-truth drug response values (scaled PRISM 1$-$AUC). For the application task, the feature is calculated with the training expression profiles and used to predict the drug response.
